# Endoplasmic Reticulum Stress (ER Stress) and Unfolded Protein Response (UPR) Occur in a Rat Varicocele Testis Model

**DOI:** 10.1155/2020/5909306

**Published:** 2020-07-29

**Authors:** Mahshid Hosseini, Erfaneh Shaygannia, Mohsen Rahmani, Anahita Eskandari, Aram Ahmadzadeh Golsefid, Marziyeh Tavalaee, Parviz Gharagozloo, Joël R. Drevet, Mohammad H. Nasr-Esfahani

**Affiliations:** ^1^Department of Reproductive Biotechnology, Reproductive Biomedicine Research Center, Royan Institute for Biotechnology, ACECR, Isfahan, Iran; ^2^Celloxess LLC, 830 Bear Tavern Road, Ewing, NJ 08628, USA; ^3^Université Clermont Auvergne, CNRS, Inserm, GReD, F-63000 Clermont-Ferrand, France

## Abstract

Using a surgically induced varicocele rat model, we show here strong evidence that the misfolded/unfolded protein response that is part of the stress response of the endoplasmic reticulum (ER) is activated in the varicocele testis (VCL), leading to the induction of apoptosis. To support this hypothesis, it is observed that the spliced variant of the X-box protein 1 (XBP1s), resulting from the activation of the inositol-requiring enzyme 1 (IRE1) membrane sensor, is significantly more represented in VCL testicular extracts. The activation of the IRE1/XBP1s pathway is also supported by the observation that the VCL testes show an increase phosphorylation of the c-Jun-kinase (JNK) known to be one intermediate of this pathway and an increased level of caspase-3, the terminal apoptotic effector, partly explaining the apoptotic status of the VCL testis.

## 1. Introduction

Varicocele (VCL) is a large varicose dilatation of the spermatic cord veins located in the bursa above and around each testicle [1]. With approximately 15% of the adult male population affected, varicocele is a relatively common disease that can occur at any age. The prevalence of varicocele in men with primary and secondary infertility is estimated to be 35% and 70-85%, respectively, making it the most common cause of male infertility [2, 3]. VCL sets in gradually and is accompanied by adverse effects on spermatogenesis, testicles, and spermatozoa. Although it is clear that higher scrotal temperature, inflammation, hypoxia, nutrient deprivation, oxidative stress, and increased apoptosis are characteristics of the varicocele testis, the full spectrum of the dysfunctional organ is not yet well defined [4]. In this complex picture, the increased generation of reactive oxygen species (ROS) recorded in the damaged and dysfunctional VCL testis and their consequences on sperm structure and function are the most documented facets [5–8].

**Figure 1:**
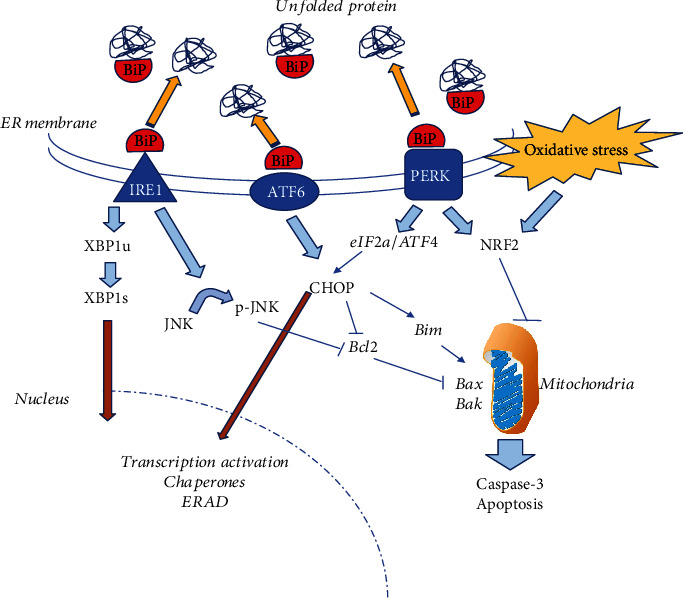
Schematic representation of the UPR/ER stress pathways. Under stress conditions (such as hyperthermia and hypoxia), unfolded proteins aggregate and accumulate in the lumen of the ER. This triggers the UPR response by driving the BiP/GRP78/HSPA5 protein, an ER chaperone, away from membrane stress sensors of the ER, including inositol-requiring enzyme 1 (IRE1), ATF6, and the PKR-like endoplasmic reticulum kinase (PERK). In short, the sequestration of BiP by unfolded proteins leads to the dimerization of IRE1, its autophosphorylation, revealing an endoribonuclease activity cleaving the mRNA of the ubiquitous X-box 1-binding protein (XBP1u). This generates a cleaved XBP1-binding protein (XBP1s) having a more potent transcriptional activating power on genes coding for more ER chaperones as well as genes involved in the ERAD response (ER-mediated apoptosis). IRE1 also triggers the phosphorylation of the c-Jun-kinase (JNK) which in turn represses the antiapoptotic Bcl2 protein, thus promoting mitochondria-dependent apoptosis signals leading to the activation of caspase-3. Similarly, sequestration of BiP by unfolded proteins in the ER causes the dimerization of PERK, which then exerts its kinase activity on eukaryotic initiation factor 2 (eIF2), resulting in increased translation of ATF4 mRNA resulting in a higher level of the C/EBP homologous protein (CHOP) itself reinforcing ER-mediated apoptosis via transcriptional activation. CHOP, in addition, will stimulate the Bim proapoptotic protein contributing to the reinforcement of mitochondria-dependent apoptosis. BiP sequestration also activates the stress-regulated transcription factor ATF6 [62] that is assumed to directly migrate to the nucleus to activate its target genes. However, recent data suggest that ATF6 acts essentially through CHOP [58]. Finally, the UPR/ER stress generates reactive oxygen species (ROS) that are known to promote apoptosis. Within the ER-stress response, PERK and ROS contribute to stimulate the antioxidant *trans*-acting factor NRF2 as a way to limit ROS proapoptotic signal.

Hyperthermia, inflammation, oxidative stress, and nutrient deprivation are situations that are also known to promote stress of the cellular endoplasmic reticulum (ER stress) characterized by the unfolded protein response (the so-called UPR/ER stress response) [9–13]. In this particular stress response, an accumulation of misfolded/unfolded proteins in the ER of defective cells is encountered, resulting in the activation of an ER-associated protein degradation pathway (ERAD) [14] which will lead to ER-mediated apoptosis [15]. In this context, the immunoglobulin binding protein (BiP), also known as the GRP78/HSPA5 protein linked to glucose (a member of the heat shock protein family), is a key player that orchestrates UPR and ER stress responses to delay cell death [15]. Briefly, and as illustrated in Figure 1, the chaperone BiP/GRP78/HSPA5 blocks the ER stress response through its interaction with ER membrane sensor proteins including PERK, IRE1, and ATF6 [14, 16, 17]. When BiP is driven out of these membrane sensors by an excess of unfolded/misfolded ER proteins, ER stress signaling begins [14] and the 3 pathways are transiently activated concurringly to adjust cell survival and apoptotic signals (see Figure 1). When the UPR response fails, as may occur under established and/or prolonged ER stress, apoptosis triggered by specific inducers such as the homologous C/EBP protein known as CHOP transcription factor, the proapoptotic factors Bim, Bax, Bak, and the Jun-kinase/caspase-3 pathway wins (Figure 1) [14–17].

In the mammalian reproductive tract, ER chaperones were shown to be important for spermatogenesis as well as for posttesticular sperm maturation [18]. In drosophila, ER stress in the male accessory organ was also reported to result in infertility [19]. In addition, the UPR/ER-stress responses were shown to occur in mammalian testis following chemical or physical stresses [20–23]. These observations prompted us to suspect that the UPR/ER stress response could be part of VCL-induced testicular dysfunction. We therefore have chosen here to evaluate markers of UPR/ER pathways in a VCL-induced rat model.

## 2. Materials and Methods

This study was approved by the Ethics Committee of Royan Institute (IR. ACECR.ROYAN.REC.1397.224), and animal care was performed according to the Animal Ethics Committee of Royan Institute recommendations.

### 2.1. Chemicals and Antibodies

The CHOP mouse antibody (Cat. No. 2895) and phospho-JNK mouse antibody (Cat. No. 9255) were purchased from Cell Signaling Technology (Danvers, MA, USA). Antibodies against glutathione peroxidase 4 (GPx4: ab125066) and nuclear factor erythroid-related transcription factor 2 (NRF2: ab62352) were purchased from Abcam (Cambridge, UK). The mouse anti-actin antibody (Cat. No: A2228), goat and rabbit immunoglobulin G (IgG) coupled with horseradish peroxidase (HRP-Cat. No: Sc-2301), and goat and mouse IgG-HRP (Cat. No: P0447) were purchased from Sigma (Saint-Louis, MI, USA), Santa-Cruz (Dallas, TX, USA), and Dako-Agilent (Santa Clara, CA, USA), respectively, while the TUNEL kit was from Promega (Madison, WN, USA). The chromomycin A3 and acridine orange dyes were purchased from Sigma (Saint-Louis, MI, USA).

### 2.2. Experimental Design and Organ/Sperm Cell Collection

Twenty 8-week-old adult male Wistar rats (weight range: 180-220 grams) were obtained from the Institute of Biotechnology (Isfahan, Iran). For the induction of varicocele, rats were anaesthetised by intraperitoneal injection of ketamine (75 mg/kg) combined with xylazine (2.5 mg/kg) according to Molina et al. [24], and VCL was induced as originally described by Ko et al. [25] and Afiyani et al. [26]. Briefly, a median incision of 2 cm was made and the diameter of the left renal vein was reduced to 1 mm; ligation of the left renal vein was performed midline at the junction of the adrenal and spermatic veins. Then, the anastomotic branch between the left testicular vein and the left common iliac vein was ligated with a 0-4 silk suture. Ten rats formed the varicocele-induced group, while ten other rats formed the control group. In this study, we did not form a sham group since on several occasions we have demonstrated that with the protocol used there were no differences between the sham group and the control group [26–28]. Rats were maintained in a controlled environment at 22-26°C, with 55-60% humidity and a 12-hour light/dark cycle. Two months after VCL induction, the body weight of the animals was measured and the animals were sacrificed as described earlier [26]. The testes were evaluated based on their morphometric characteristics, including length, width, weight, thickness, and volume. The left testicle of each rat was divided into two parts; one part was fixed in a Bouin solution for histopathological evaluation while the other part was cryopreserved and stored prior to evaluation of the genes, proteins and molecules of interest.

The epididymides were also collected and divided into three parts. Sperm cell collection for evaluation of structural and functional spermatozoa parameters (sperm count, motility, morphology, lipid peroxidation, DNA damage, histone, and protamine content) was performed on epididymal tails (cauda) after chopping the tissue into small pieces which were then incubated at 37°C for 30 minutes in 1 ml VitaSperm medium (Inoclon, Iran).

### 2.3. Histological Analyses

Testicular sections fixed by Bouin were evaluated after staining with haematoxylin/eosin (HE) and parameters such as mean percentage of tubular diameter, epithelial height, meiotic index, percentage of spermatogenesis, and Johnsen's score [29] were evaluated using an Olympus CX31 microscope (40x magnification). The TUNEL stain was used as previously indicated for the evaluation of apoptosis [30].

### 2.4. Spermatozoa Structural and Functional Evaluation

**Figure 2:**
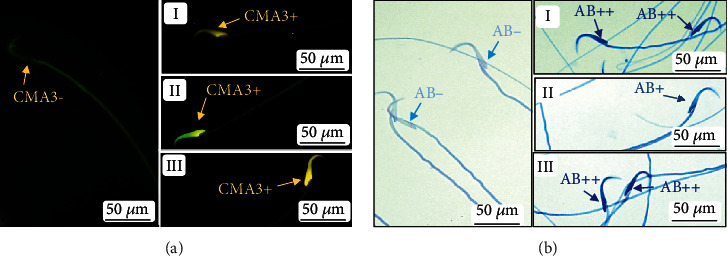
Images of chromomycin A3 (CMA3) and aniline blue (AB) staining for assessment of sperm protamine deficiency and persistent histone, respectively. (a) Sperm with normal protamine content or CMA3 negative, I-III: sperm with different intensities of protamine deficiency or CMA3 positive. (b) Sperm with normal persistent histone or AB negative, I-III: sperm with different degrees of abnormal persistent histone (AB+ and AB++).

For the evaluation of spermatozoa mobility, 10 *μ*l of sperm cell samples retrieved from the cauda epididymides (see above) were smeared on a slide and observed using an optical microscope (CX31 Olympus, Dubai, EAU). For each animal, at least 200 sperm cells were evaluated from different fields, and the mean percentage of mobile sperm was recorded. Sperm counts were estimated using a Makler counting chamber (sperm meter; sperm processor, Garkheda, Aurangabad, India) using a LABOMED CxL optical microscope (magnification: 20x) and data were expressed as million cells per ml. For morphology, 50 *μ*l of cauda-retrieved spermatozoa samples were stained using eosine/nigrosine according to Afiyani et al. [26]. The percentages of abnormal sperm morphology presented for each sample include abnormal head, neck, and flagellum. Sperm membrane lipid peroxidation level was monitored using BODIPY 581/591 C11 (Cayman Chemicals, Ann Arbor, MI, USA) as described earlier by Aitken et al. [31]. Briefly, 2 million sperm cells were mixed with the BODIPY probe (5 mM final concentration) in the presence (positive control) or absence of H_2_O_2_ (10 mM). Controls and test tubes were then incubated for 30 min at 37°C. Samples were then washed twice using a phosphate-buffered saline (PBS) solution (1X) at 2000 rpm for 5 min at room temperature. A flow cytometer (FACSCalibur, Becton Dickinson, San Jose, CA, USA) was used to assess the percentage of sperm cells showing lipid peroxidation. For sperm DNA damage, the acridine orange (AO) staining method was used [32]. Aliquots of 20 *μ*l of cauda-retrieved sperm cells were smeared on slides and fixed with Carnoy's solution at 4°C overnight. Then, the slides were stained using 150 *μ*l of a freshly prepared AO solution (in citric acid 0.1 M, NaH2PO4 0.3 M, pH: 2.5) for 90 min in the dark at room temperature. The slides were then washed with PBS (1X) twice and observed using a fluorescent microscope (OPTIKA, Ponteranica, Italy). Two hundred sperm cells were counted on each slide, and the percentage of DNA damaged cells (cells having a red/orange nucleus) was calculated. For sperm chromatin integrity, both aniline blue (AB) and chromomycin A3 (CMA3) staining were used. AB indirectly evaluates the level of condensation of the sperm nucleus via its affinity to lysine-rich histones while CMA3 competes with protamines [33, 34]. To perform the AB staining, washed sperms were fixed with 3% glutaraldehyde in 0.2 M phosphate buffer (pH = 7.2) for 2 hours at 4°C. Then, two smears were prepared and air-dried for each condition. Aqueous AB (5%) in acetic acid (4%) was added to the slides for 5 minutes and the slides were washed once with phosphate-buffered saline (PBS 1X). The slides were observed using an optical microscope (OPTIKA, Ponteranica, Italy, magnification 100x). For each slide, 200 spermatozoa were counted, and the percentage of spermatozoa with immature chromatin (dark blue stained nucleus) was calculated (Figure 2). To perform the CMA3 staining, 30 *μ*l washed sperm were fixed with 30 *μ*l Carnoy's solution for 5 minutes at 4°C. The slides were then rinsed with PBS (1X) and stained with CMA3 solution (0.25 mg/ml). The slides were then rinsed and covered. For each slide, 200 spermatozoa were counted using a fluorescent microscope (OPTIKA, Ponteranica, Italy, magnification 100x), and the percentage of CMA3-positive spermatozoa was calculated (Figure 2).

### 2.5. Total RNA Extraction and RT-PCR Analyses

In short, 50 mg of frozen testis tissues was used to extract RNA using the TRIzol reagent (Thermo Fisher Scientific, Waltham, MA, USA) according to the manufacturer's protocol. RNA quantification was done by evaluating the absorbance ratio A260/A280. Complementary DNA was prepared using a commercial reverse transcription kit (Takara Bio Inc., Shiga, Japan). The qRT-PCR were performed in a total volume of 10 *μ*l containing 1 *μ*l cDNA, 2.8 *μ*l H2O, 0.2 *μ*l Rox, 1 *μ*l specific primers, and 5 *μ*l SYBR Green using a StepOnePlus thermal cycler (Applied Biosystems, Abi, Foster City, CA, USA). The 2^-*ΔΔ*CT^ method was used for data analysis. Primers were designed using the software Beacon Designer 7 and are presented in Table 1.

### 2.6. Western Blot Analyses

Testicular protein extracts (30 *μ*g) were loaded on 12% SDS PAGE gel subsequently transferred on a PVDF membrane (Merck, Darmstadt, Germany). The membranes were then blocked and incubated with the different primary antibodies including: anti-phospho-SAPK/JNK (dilution: 1/1000), anti-GADD153/CHOP (dilution: 1/2000), anti-GPx4 (dilution: 1/200), anti-*β*-actin (dilution: 1/500), and anti-NRF2 (dilution: 1/500) overnight at 4°C. The membranes were then washed 3 times and appropriate secondary antibodies (anti-rabbit/anti-goat/anti-mouse IgG-HRP conjugated) depending on the primary antibody were used for 2 hours at room temperature. The ECL detection kit (Thermo Fisher Scientific, Waltham, MA, USA) was then used following the supplier recommendation. The *β*-actin signal was used to normalize sample loading differences. The ImageJ software (version 1.42q) was used to quantify the different signals.

### 2.7. Statistical Analysis

The statistical analysis was performed using IBM SPSS Statistics 25.0 and GraphPad Prism (version 7.00) for graphic design (GraphPad Software, San Diego, California, USA). The mean ± SEM was represented for all data. An independent *t*-test was used to compare the study tests between the control and varicocele groups.

## 3. Results

### 3.1. Testis and Spermatozoon Characteristics in VCL-Induced Rats

The induction of VCL was first evaluated in the group of induced animals. The VCL induction protocol did not significantly alter the physiology of the animals, as evidenced by the fact that the mean body weight of the VCL and control animals was not significantly different (Table 2). As expected in a situation of VCL, the left testis volume reduction was found to be significant (Table 2). As expected also, and consistent with previous data, Table 3 shows that VCL induction resulted in a decrease in mean testicular tubule diameter and epithelium height associated with a decrease in the meiotic index, the percentage of spermatogenesis, and the Johnsen score.

**Figure 3:**
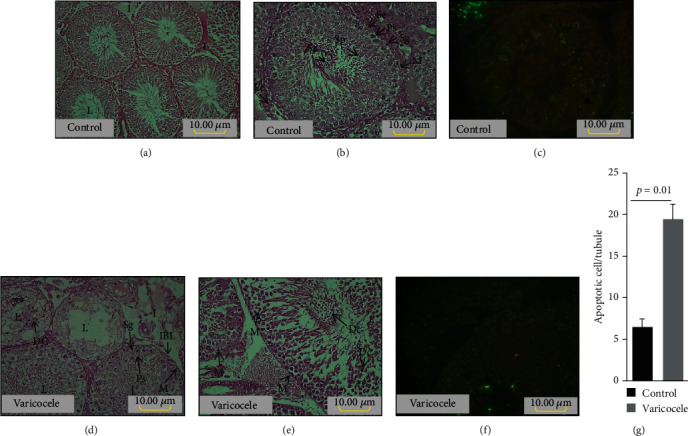
H&E staining in the left seminiferous tubules of control (a, b) and varicocele (d, e) groups. L: lumen. I: inter seminiferous connective tissue. Se: Sertoli cell. Ps: primary spermatocyte. Sp: secondary spermatocyte. Sg: spermatogonia. M: myoid cell. Sz: spermatozoa. Dc: degraded cell. Le: Leydig cell. IBL: irregular basal lamina. The two asterisks (∗∗) in panels (d, e) point out at intracellular vacuoles. Representative TUNEL staining of left testis sections of control (c) and varicocele (f) groups. Arrows show the TUNEL^+^ germ cells. The graph on the right (g) show comparison of mean TUNEL^+^ germ cells between control and varicocele groups (*n* = 3 each). Independent *t*-test was used for comparison two groups. Data was expressed as mean ± SEM.

**Figure 4:**
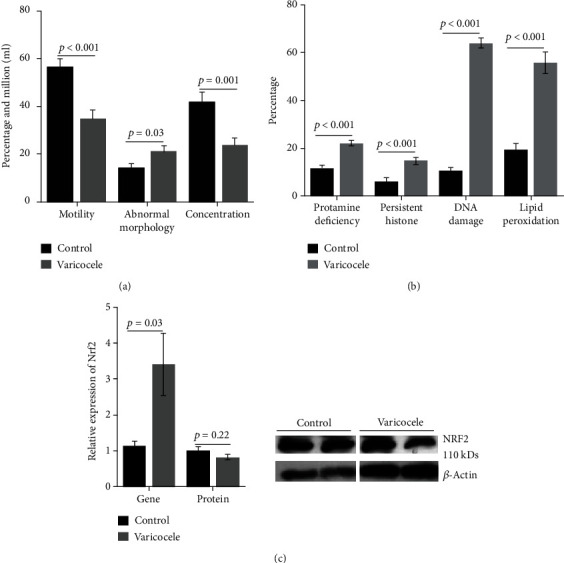
Bar graphs showing control vs. VCL comparisons for selected sperm parameters including sperm motility, sperm abnormal morphology, and spermatozoa concentration (a) as well as protamine content, histone content, DNA damage, and lipid peroxidation (b). In graph (c), the relative NRF2 mRNA and protein content of control vs. VCL testis extracts are shown. *n* = 10 in each group. Independent *t*-test was used for comparison between the two groups. Data are expressed as mean ± SEM. Nota bene: In graph A, the *y*-axis numbers correspond to percentages for motility and abnormal morphology while it corresponds to sperm cell concentrations (in million cells/ml) for sperm counts.

Figures 3(a)–3(f) illustrate the classic structural defects observed in VCL testes, including the destructuring of the seminiferous epithelium, vacuolization, increased connective tissue, and Sertoli and Leydig cell abnormalities. In addition, Figure 3(g) shows that VCL testicular sections contain significantly more apoptotic cells than control testicular sections. Considering the effect of VCL induction on sperm parameters, we show in Figure 4(a) that the mean sperm concentration and motility were significantly decreased in semen samples taken from the tail of the epididymis of VCL animals. At the same time, we recorded a small but significant increase (*p* = 0.03) in abnormal sperm morphologies in VCL animals. Upon closer examination of the integrity of the sperm nucleus in VCL animals, we recorded clear situations of protamine deficiency associated with increased levels of persistent histones and a very significant increase in sperm DNA damage, as shown by acridine orange staining (Figure 4(b)). These abnormalities reflecting spermatozoa nuclear immaturity and/or damage were also accompanied by an increased level of peroxidation of sperm membrane lipids, as shown by the higher BODIPY reactivity of VCL sperm samples (Figure 4(b)).

To assess the well-known situation of oxidative stress associated with VCL, we used real-time qPCR to compare control and VCL testes. We show in Figure 4(c) that the accumulation of NRF2 mRNA (a major transcription factor orchestrating the antioxidant response under oxidative stress) is significantly increased in VCL testes (3.41 ± 0.86) compared to control testes (1.14 ± 0.13) (*p* = 0.03). However, the increased accumulation of NRF2 mRNA in the VCL testes does not result in an increase in NRF2 protein content; this will be discussed later.

### 3.2. Assessment of the UPR/ER-Stress and Apoptotic Pathways in VCL Testis

**Figure 5:**
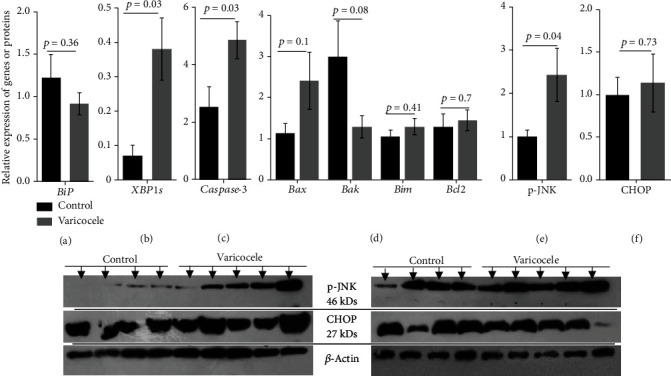
Comparisons of testicular markers involved in the UPR/ER stress pathways between control and VCL groups. (a) BiP mRNA accumulation. (b) IRE1/XBP1s mRNA accumulation (c, d). mRNA levels of proapoptotic caspase-3, Bax, Bak, and Bim (c) and antiapoptotic Bcl2 (d). (e) IRE1-mediated p-JNK protein levels. (f) CHOP protein levels. Independent *t*-test was used for comparison of these parameters between two groups. Data are expressed as mean ± SEM.

To evaluate the induction of UPR/ER stress pathways in VCL testes, we first chose to monitor the level of the ER chaperone protein BiP/GRP78/HSAP5 using a qRT-PCR approach. We found no difference in the accumulation of BiP transcripts when we compared VCL testes extracts with control extracts (Figure 5(a)). We then examined the spliced form of the XBP1s protein, the transcription factor that performs UPR [35]. As shown in Figure 1, the splicing of XBP1 mRNA is one of the benchmarks of UPR/ER stress response via the IRE1 membrane-associated protein [36]. IRE1 oligomerizes and activates its ribonuclease domain by autophosphorylation. It then catalyzes the excision of a 26 bp intron on the mRNA of the ubiquitously expressed XBP1 protein (XBP1u). This causes a shift in the XBP1s coding sequence, resulting in the translation of a distinct XBP1s protein. Using this specific feature, we show in Figure 5(b) that the spliced form of the XBP1 transcript (XBP1s) is 6 times more represented in VCL testis extracts than in control testis extracts (0.37 ± 0.09 versus 0.06 ± 0.02; *p* = 0.03), confirming the activation of the UPR pathway in the rat testis of VCL.

As indicated above (Figure 1), when the UPR response is prolonged, IRE1 stimulates the JNK/p-JNK pathway which induces apoptosis [37] via, in particular, downregulation of the antiapoptotic protein Bcl2 and upregulation of mitochondrial-associated proapoptotic actors including Bax and Bak, leading to induction of caspase-3. We show in Figure 5(c) that in the VCL testis, the JNK pathway is activated as revealed by the increased level of phospho-JNK (*p* = 0.04). We also show in Figure 5(d) that caspase-3 transcript accumulation is significantly greater in the VCL testis compared to controls (4.84 ± 0.64 versus 1.14 ± 0.14, *p* = 0.03). To further evaluate the apoptotic network in the VCL testis, we also monitored the accumulation of two classical proapoptotic factors associated with mitochondria, Bax and Bak (Figure 5(e)). As shown in Figure 5(d), the accumulation of Bax and Bak mRNA did not increase significantly in the VCL testis.

Apart from the proapoptotic IRE1/JNK pathway, the accumulation of unfolded/misfolded proteins in the ER has the potential to trigger the PERK/ATF4/CHOP pathway, which should contribute to proapoptotic signals (see Figure 1). We show in Figure 5(f) that VCL testicular extracts did not show a higher CHOP content when compared to control testis.

## 4. Discussion

To test whether VCL testis could be concerned by UPR/ER stress mechanisms, we used our expertise in VCL-induced rat models [24–28]. After verifying that our VCL-induced animals had all the classical characteristics expected in terms of testicular damage and sperm distorted parameters [28, 38–40], we then focused our research on marker characteristics of UPR/ER stress pathways.

Although BiP/GRP78 expression was shown to be modulated in the rodent testis throughout spermatogenesis [41], in our rat model of surgically induced VCL, we show that there is no difference in testicular BiP content. There might be a rationale for that observation as it was shown that the expression level of BiP is mainly regulated at the posttranscriptional level through the control of protein degradation processes that may readily allow BiP to return to optimal levels [42]. Nevertheless, despite this observation that could suggest that there is no-ER-stress response ongoing in the VCL testis, we further investigated the downstream effectors of the UPR/ER stress response starting with the XBP1s spliced variant mediated by IRE1 activation [43, 44]. We clearly show that the VCL testis extracts contain a large excess of XBP1s, which is indicative of the activation of the IRE1 membrane sensor. Our observation that the downstream proapoptotic JNK/phospho-JNK switch [45, 46] is also mobilized, as shown by the increased representation of phospho-JNK in testicular VCL extracts, confirms the activation of the IRE1 ER sensor. Indeed, it was reported that if processing of XBP1 mRNA by the RNase domain of IRE1*α* promotes survival of ER stress, activation of the mitogen-activated protein kinase JNK by IRE1 late in the ER stress response promotes apoptosis [47]. Consistent with the activated IRE1 proapoptotic pathway in VCL testes, we logically found an increased level of caspase-3, which partly explains the apoptotic observations made on sections of VCL testes via the TUNEL assay (Figure 3). Positive regulation of caspase-3 in the rat VCL testis has been reported previously [48]. Similarly, studies have reported IRE1-mediated apoptosis in germ cell lines following various stresses [22, 49–51]. Our data corroborates also earlier data reported elsewhere [52].

The second pathway by which UPR/ER stress can modulate apoptosis is the PERK/ATF4/NRF2/CHOP pathway. On the one hand, PERK activity induces the expression of the homologous protein of the C/EBP transcription factor (CHOP) (see Figure 1). We show here that the CHOP-dependent PERK pathway does not appear to be preponderant because CHOP levels do not change in the VCL testis. Consistent with the observation that CHOP level remains unchanged, neither Bim nor Bcl2 levels were changed in the VCL testis (Figure 5(e)). Normally, in a situation where CHOP is activated, one would expect downward regulation of antiapoptotic Bcl2 and upward regulation of proapoptotic Bim proteins [53]. None of these events occur in the VCL testis. In addition, the mitochondrial-associated proapoptotic effectors, Bax and Bak, which are expected to partially respond to Bim, were not found to be altered in the VCL testis. On the other hand, PERK activity allows the nuclear translocation of NRF2 where it increases the expression of antioxidant genes [10]. We cannot answer the question of whether there is more nuclear NRF2 in VCL testis cells because we have not specifically followed up on this question. We only show that in VCL testis extracts, we record a transcriptional induction of NRF2 that was not accompanied by an increase in NRF2 protein content or positive regulation of a NRF2 target gene (the glutathione peroxidase 4: GPx4 gene, not shown). This is rather difficult to explain, although a similar situation has been reported in chronic liver disease UPR/ER stress response where an increase in XBP1s has been shown to be associated with increased NRF2 degradation [54]. Therefore, it is possible that the recorded increase in XBP1s in the VCL testes may mask the increase in NRF2 that would have been expected otherwise.

The ATF6 channel is the third branch through which it is commonly accepted that the UPR/ER stress response is mediated [55]. Again, the sequestration of BiP/GRP78/HSPA5 by misfolded/unfolded ER proteins activates the ATF6 transcription factor. Early activation of ATF6 in the ER-stress response is expected in return to activate the transcription of BiP providing more chaperone aiming at alleviating the ER stress [56]. It was recently reported that ATF6 exerts also its action through CHOP which, in turn, regulates the decision on the fate of the cells [57]. Since neither BiP nor CHOP expression was shown upregulated in our model of VCL testis, at the time we evaluated the tissue response, it may suggest that we are probably late in the UPR/ER stress response kinetics as hypothesized elsewhere [9, 58, 59].

In conclusion, we show here that the UPR/ER response is most likely activated in the VCL testis as evidenced by the engagement of the IRE1/JNK/XBP1s pathway. In addition, caspase-3-mediated apoptosis, which is known to be the ultimate response to UPR/ER stress, is also activated in the VCL testis. The observation that the PERK/ATF4/ATF6/CHOP pathway does not appear to be activated in the VCL testis can be explained. Several authors have recently shown that the 3 pathways known to mediate the stress response of the UPR/ER work with a specific kinetics, with PERK being the first to be activated, then ATF6, and finally IRE1 [9, 58, 59]. Moreover, it is proposed that when IRE1 is activated, the XBP1s variant has been shown to exert negative control over the PERK channel. Therefore, our interpretation of the situation in the VCL testis rat model we studied is that we are likely in the late stages of the UPR/ER stress response as it is strongly suggested by the activation of the p-JNK-induced caspase-3 apoptotic signal. Consistent with our data and interpretation is the very recent publication that the ER stress markers p-JNK, p-IRE, cleaved-caspase-3, and Bax are induced in rat VCL testes, a situation that could be reversed by oral administration of anti-inflammatory and antioxidant herbal compounds [58]. Our data also support Kim et al. [44], who indicated that IRE1 is the primary mediator of the stress response of the UPR/ER in testicular hyperthermia in mice. As already suggested by the recent work of Karna et al., the data collected so far in animal VCL models opens the way for additional therapeutic actions to reduce the impact of human VCL by means of antioxidant and anti-inflammatory molecules [61].

## Figures and Tables

**Table 1:** List of primers used for real-time RT-PCR analyses.

Gene	GenBank accession nb.	Primer sequence (5′-3′) F: forward; R: reverse	Amplicon size (bp)
*Grp78/BiP/HSPA5*	NM_013083.2	F *TAACAATCAAGGTCTACGAAGG* R *CCATTCACATCTATCTCAAAGGT*	193
*XBP1s*	NM_001271731.1	F *CTGAGTCCGCAGCAGG* R *CTTGTCCAGAATGCCCAAAAGG*	119
*XBP1u*	NM_001004210.2	F *GTCCGCAGCACTCAGACTAC* R *CTGGGGAAGGACATTTGAAAAAC*	178
*NRF2*	NM_031789.2	F *TGCCATTAGTCAGTCGCTC* R *GTGCCTTCAGTGTGCTTC*	99
*Bcl2*	NM_016993.1	F *ACTTCTCTCGTCGCTACCGTC* R *AAGAGTTCCTCCACCACCGT*	106
*Caspase-3*	NM_012922.2	F *CGGTATTGAGACAGACAGTGGAAC* R *GCGGTAGAGTAAGCATACAGGAAG*	90
*Bax*	NM_017059.2	F *GGATCGAGCAGAGAGGATGG* R *ACACTCGCTCAGCTTCTTGG*	91
*Bak*	NM_053812.1	F *CAGAGAGGTGGTTGGGTGG* R *GTGGGTTGGGGAGAGGTTTAG*	181
*Bim*	NM_171988.2	F *CACAAACCCCAAGTCCTCC* R *AGTCTCATTGAACTCGTCTCC*	152
*GAPDH*	NM_017008.4	F *TGCCGCCTGGAGAAACC* R *TGAAGTCGCAGGAGACAACC*	121

**Table 2:** Body weight and testis volume in control and VCL animals.

Parameters	Control	Varicocele
Body weight (gm)	340.25 ± 8.89	325.50 ± 16.73
Left testis volume (cm)	1.43 ± 0.05^a^	1.09 ± 0.08^a^

Data are represented as mean ± SEM, and superscript letters represent significant differences at *p* < 0.05 probability level.

**Table 3:** testis histological and spermatogenetic parameters.

Parameters	Control	Varicocele
Tubular diameter (*μ*m)	9.50 ± 0.30^a^	8.40 ± 0.5^a^
Epithelium height (*μ*m)	3.49 ± 0.15^b^	2.5 ± 0.11^b^
Meiotic index	78.5 ± 2.95^c^	43.26 ± 5.40^c^
Spermatogenesis (%)	96.44 ± 2.67^d^	41.30 ± 4.36^d^
Johnsen score	9.62 ± 2.7^e^	5.5 ± 2.57^e^

All data are represented as mean ± SEM and superscript letters represent significant differences at *p* < 0.05 probability level.

## Data Availability

All reported data (with the exception of the GPx4 mRNA accumulation) are presented in the manuscript.
